# Novel Reclassification of Adult Diabetes Is Useful to Distinguish Stages of β-Cell Function Linked to the Risk of Vascular Complications: The DOLCE Study From Northern Ukraine

**DOI:** 10.3389/fgene.2021.637945

**Published:** 2021-07-02

**Authors:** Olena Fedotkina, Oksana Sulaieva, Turkuler Ozgumus, Liubov Cherviakova, Nadiya Khalimon, Tetiana Svietleisha, Tetiana Buldenko, Emma Ahlqvist, Olof Asplund, Leif Groop, Peter M. Nilsson, Valeriya Lyssenko

**Affiliations:** ^1^Department of Clinical Science, Center for Diabetes Research, University of Bergen, Bergen, Norway; ^2^Lund University Diabetes Center, Department of Clinical Sciences, Lund University, Skåne University Hospital, Malmö, Sweden; ^3^Medical Laboratory CSD, Kyiv, Ukraine; ^4^Chernihiv Regional Hospital, Chernihiv, Ukraine; ^5^City Hospital Mo. 2, Chernihiv, Ukraine; ^6^City Hospital No., Chernihiv, Ukraine; ^7^Department of Health Care of Chernihiv Regional State Administration, Chernihiv, Ukraine

**Keywords:** clustering, β-cell function, diabetes complications, genetics, adult diabetes

## Abstract

**Background:**

Presently, persons with diabetes are classified as having type 1 (T1D) or type 2 diabetes (T2D) based on clinical diagnosis. However, adult patients exhibit diverse clinical representations and this makes treatment approaches challenging to personalize. A recent Scandinavian study proposed a novel classification of adult diabetes into five clusters based on disease pathophysiology and risk of vascular complications. The current study aimed to characterize new subgroups of adult diabetes using this strategy in a defined population from northern Ukraine.

**Methods:**

We analyzed 2,140 patients with established diabetes from the DOLCE study (*n* = 887 with new-onset diabetes and *n* = 1,253 with long duration). We used the k-means approach to perform clustering analyses using BMI, age at onset of diabetes, HbA_1c_, insulin secretion (HOMA2-B), and insulin resistance (HOMA2-IR) indices and glutamic acid decarboxylase antibodies (GADA) levels. Risks of macro- (myocardial infarction or stroke) and microvascular [retinopathy, chronic kidney disease (CKD) and neuropathy] complications and associations of genetic variants with specific clusters were studied using logistic regression adjusted for age, sex, and diabetes duration.

**Results:**

Severe autoimmune diabetes (SAID, 11 and 6%) and severe insulin-deficient diabetes (SIDD, 25 and 14%) clusters were twice as prevalent in patients with long-term as compared to those with new-onset diabetes. Patients with long duration in both SAID and SIDD clusters had highest risks of proliferative retinopathy, and elevated risks of CKD. Long-term insulin-resistant obese diabetes 1 (IROD1) subgroup had elevated risks of CKD, while insulin-resistant obese diabetes 2 (IROD2) cluster exhibited the highest HOMA2-B, lowest HbA_1c_, and lower prevalence of all microvascular complications as compared to all other clusters. Genetic analyses of IROD2 subgroup identified reduced frequency of the risk alleles in the *TCF7L2* gene as compared to all other clusters, cumulatively and individually (*p* = 0.0001).

**Conclusion:**

The novel reclassification algorithm of patients with adult diabetes was reproducible in this population from northern Ukraine. It may be beneficial for the patients in the SIDD subgroup to initiate earlier insulin treatment or other anti-diabetic modalities to preserve β-cell function. Long-term diabetes cases with preserved β-cell function and lower risk for microvascular complications represent an interesting subgroup of patients for further investigations of protective mechanisms.

## Introduction

Diabetes represents a global health problem, affecting today more than 400 million people worldwide, which is estimated to increase up to 600 million by 2030 (IDF, 2019). Diabetes in adults, of which type 2 diabetes (T2D) being the most common type, comprises around 90% of all diabetes cases. The incidence and prevalence of adult diabetes are more rapidly increasing in the low- and middle-income countries (IDF, 2019). The official prevalence of diabetes in Ukraine was reported to rise by about 25% from 2007 to 2019, reaching 8.4% in 2019 (Khalangot and Tronko, 2007; IDF, 2019). This situation is particularly alarming because nearly an equal number of people have undiagnosed diabetes in Ukraine meaning that the prevalence rate is doubled in real life (IDF, 2019; Mankovsky, 2020). Diabetes is one of the leading causes of blindness, end-stage renal disease, limb amputation, heart disease, stroke, liver cirrhosis, and premature death in working age adults (IDF, 2019). Numerous clinical and genetic studies demonstrated that adult diabetes is a highly heterogeneous metabolic disease with diverse underlying mechanisms (Tuomi et al., 2014; McCarthy, 2017). Lack of pathophysiology-based classification hampers personalized therapy targeting specific complications of diabetes (Fitipaldi et al., 2018). This shortcoming was recently addressed in a Scandinavian cohort of patients with newly diagnosed adult diabetes, by applying an unbiased unsupervised clustering approach to propose a reclassification of the disease (Ahlqvist et al., 2018). Based on six clinically affordable parameters including glycated hemoglobin (HbA_1c_), body-mass index (BMI), age at diabetes onset, insulin resistance, insulin secretion calculated using homeostasis model assessment (HOMA2), and glutamic acid decarboxylase antibody (GADA) levels, a new five-cluster classification scheme for adult diabetes was proposed. These clusters reflected essential pathophysiological mechanisms in the disease and were named accordingly as severe autoimmune diabetes (SAID), severe insulin-deficient diabetes (SIDD), severe insulin-resistant diabetes (SIRD), mild obesity-related diabetes (MOD), and mild age-related diabetes (MARD). This novel classification has now been validated in a number of studies across Europe and Asia, emphasizing the robustness of the approach (Dennis et al., 2019; Zaharia et al., 2019; Zou et al., 2019) even though there was some ethnic heterogeneity reported in an Asian-Indian study (Anjana et al., 2020). The suggested clustering approach is attractive for clinical practice because of the opportunity to predict the risks of diabetic complications at diagnosis and tailor patients’ therapy accordingly (Ahlqvist et al., 2018; Zaharia et al., 2019).

The aim of the present study was to assess the prognostic value of the new reclassification approach by Ahlqvist et al. (Ahlqvist et al., 2018) for the first time in an East-European population from Ukraine including patients with newly diagnosed and long-term adult diabetes.

## Participants and Methods

### Study Cohort

The Diagnostic Optimization and Treatment of Diabetes and its Complications in the Chernihiv Region, Ukraine (DOLCE) is a hospital- and primary health care-based study of individuals with diabetes and their healthy relatives, including total of 6,095 participants. The cohort consists of 785 persons with T1D, 4,297 with T2D, 62 with unspecified diabetes, and 951 healthy first- or second-degree relatives. All participants completed a questionnaire supervised by an endocrinologist and a trained diabetes nurse, which covered the person’s medical history and included information of family history of diabetes, anthropometric measurements (weight, height, and blood pressure), alcohol intake, smoking, diabetes medication, antihypertensive and lipid-lowering treatments. Information of prevalent cardiovascular events, neuropathy, chronic kidney disease, and stages of retinopathy was reported by primary care physicians using patients’ hospital discharge records as primary source and was used as data entry into the DOLCE database at the screening visit. Fasting blood samples were drawn for plasma glucose and HbA_1c_ measurements. Additional plasma and serum samples were stored at –80°C for C-peptide, insulin, lipids, and glutamic acid decarboxylase antibodies (GADA) measurements, which were performed at the Department of Clinical Chemistry, Scania University Hospital, Malmö, Sweden. A written informed consent form was obtained from every participant. The DOLCE study was approved by the local ethics committees (approval number for Ukraine Dnr17/2011–09–14; for Norway 2019/28968).

### Measurements and Calculations

C-peptide concentrations were measured with an electro-chemiluminescence immunoassay on Cobas e411 (Roche Diagnostics, Mannheim, Germany) or a radioimmunoassay (Human C-peptide RIA; Linco, St Charles, MO, United States; or Peninsula Laboratories, Belmont, CA, United States). GADA were measured with an ELISA from the samples collected at the screening visit. Test results greater than or equal to 5 U/ml were considered as positive. The radio binding assays had 62–88% sensitivity and 91–99% specificity, and the ELISA assay had 72% sensitivity and 99% specificity (Combinatorial Autoantibody or Diabetes/Islet Autoantibody Standardization Programs 1998–2013). β-cell function (HOMA2-B) and insulin resistance (HOMA2-IR) were assessed with Homoeostasis Model Assessment 2 (HOMA2) and were calculated with the HOMA2 calculator using C-peptide and fasting glucose measurements (Levy et al., 1998; HOMACalculator, 2020). Data values for BMI, HOMA2-B, and HOMA2-IR with more than three standard deviations were excluded. Only individuals with age at diabetes diagnosis older than 18 years and complete information on BMI, age at onset, HbA_1c_, HOMA2-B, HOMA2-IR, sex, duration of diabetes, and complication status were included in the final analysis (*n* = 2,140).

### Definition of Diabetic Complications

Proliferative diabetic retinopathy (PDR) was defined as having one of the following conditions: (a) proliferative retinopathy, (b) laser treatment, or (c) blindness of either one or both of the eyes. Stages of PDR were based on fundus photographs and were evaluated by ophthalmologists. Chronic kidney disease (CKD) was defined as having at least one of the following conditions: (a) estimated glomerular filtration rate less than 60 mL/min/1.73 m^2^ (eGFR < 60), (b) clinically documented diagnosis of nephropathy, dialysis, or end-stage renal disease (ESRD). eGFR was calculated using Modification of Diet in Renal Disease (MDRD) formula as: 186 × (Creatinine/88.4)^–1.154^ × (age)^–0.203^ × 0.742 for female participants; 186 × (Creatinine/88.4)^–1.154^ × (age)^–0.203^ for male participants (Mula-Abed et al., 2012). Neuropathy was defined as clinically diagnosed peripheral neuropathy. Cardiovascular diseases were defined as either presence of myocardial infarction or stroke using International Classification of Diseases (ICD)-10 codes I21, I24, and I61–I64, respectively.

### Statistical Analysis

In SAID cluster we included individuals with positive GADA. SIDD, SIRD, MOD, and MARD cluster analysis was performed on individuals who were negative for GADA antibodies (where GADA measurements were available) using previously defined parameters by Ahlqvist et al. (Ahlqvist et al., 2018): HbA_1c_, BMI, age at onset of diabetes, HOMA2-B, and HOMA2-IR. The analyses were done in two groups of patients: (i) long-term diabetes with more than 3 years of disease duration and (ii) new-onset diabetes with less than 3 years of disease duration to harmonize with the Swedish ANDIS cohort employed as the reference (Ahlqvist et al., 2018). The analyses were performed using k-means clustering on the centered and scaled values. We used “kmeansruns” function from R package fpc v2.2–8 with 4 as the assigned number of clusters and default parameters: krange = 4, criterion = “asw,” iter.max = 100, runs = 100, alpha = 0.001, critout = FALSE (Hennig, 2020). Male and female patients were analyzed separately to avoid sex bias, and results were merged afterward. Risks for diabetic complications in each cluster were calculated using logistic regression adjusted for age, sex, and diabetes duration with the MARD cluster used as reference. To adjust for multiple phenotype testing, we calculated a false discovery rate (FDR). FDR was calculated using p.adjust function implemented in the “stats” package in R (R Core Team, 2021). FDR < 0.05 was considered statistically significant.

### Genetics

Genotyping of DNA samples was available only in patients with clinically defined T2D using Infinium Core Exome Chip InfiniumCoreExome-24v1-1 (https://www.illumina.com). Imputation was done using Michigan Imputation Server and Reference Panel HRC r1.1 2016. Standard quality control steps for Genome-Wide Association Studies (GWAS) were applied (Marees et al., 2018). We analyzed SNPs associated with T2D, which were previously reported in the DIAGRAM GWAS meta-analysis (Morris et al., 2012). Logistic regression adjusted for sex and age was used to study associations between the insulin resistant obese diabetes 2 cluster and the genetic variants as compared with all other clusters in patients with long-term diabetes. SAID cluster was not included in the genetic analysis due to small sample size of individuals with genotypes in this group (*n* = 26). Bonferroni correction was used to adjust for multiple testing in genetic association tests. *P* < 0.05 was considered statistically significant.

## Results

The clinical characteristics and prevalence of macro- and microvascular complications in adult patients with (a) new-onset and with (b) long-term diabetes are shown in Table 1.

**TABLE 1:** Clinical characteristics of patients with adult diabetes in the DOLCE study.

Phenotype	New-onset	Long-term
N (men,%)	887 (36%)	1,253 (34%)
Age at visit, years	56.9 (12.5)	61.2 (10.0)
Age at onset of diabetes, years	55.8 (12.4)	50.1 (10.8)
Diabetes duration, years	1.1 (1.1)	11.0 (6.9)
HbA_1c_,%	7.9 (2.1)	9.0 (2.0)
HbA_1c_, mmol/mol	62.9 (23.4)	74.4 (21.3)
BMI, kg/m^2^	30.7 (5.6)	30.9 (5.3)
Waist, cm	97.4 (14.3)	99.1 (12.7)
HOMA2-B	81.1 (44.6)	63.9 (45.9)
HOMA2-IR	2.4 (1.2)	2.2 (1.3)
C-peptide, nmol/l	0.9 (0.5)	0.8 (0.5)
Without treatment,%	46.9%	12.1%
Tablets,%	42.6%	51.6%
Insulin,%	8.8%	27.7%
Tablets and insulin,%	1.7%	8.5%
Sulfonylurea,%	27%	45%
PDR,%	0.7%	5.5%
CKD,%	12.4%	26.7%
Neuropathy,%	34.3%	85.6%
CVD,%	8.6%	14.9%

**Data are represented as mean ± (SD). HOMA2-B, homoeostatic model assessment 2 estimates of β-cell function; HOMA2-IR, homoeostatic model assessment 2 estimates of insulin resistance; PDR, proliferative diabetic retinopathy; CKD, chronic kidney disease; CVD, cardiovascular disease.**

### New-Onset Adult Diabetes

There were 887 (36% men) with new-onset diabetes (years, 1.1 ± 1.1). Oral glucose-lowering treatment was reported for 42.6% of the patients, while insulin treatment was initiated in 8.8% of the patients. The prevalence of PDR was 0.7%, and CKD was 12.4%. Peripheral neuropathy was reported in 34.3%, while non-fatal CVD occurred in 8.6% of the individuals (Table 1).

### Long-Term Adult Diabetes

There were 1,253 persons (34% men) with long diabetes duration (mean ± SD, years, 11.0 ± 6.9). BMI in this group was similar to the new-onset diabetes group (kg/m^2^, 30.9 ± 5.3 and 30.7 ± 5.6). Oral glucose-lowering treatment was reported for 51.6% of the patients, while insulin treatment was initiated in 27.7% of the patients. Glycemic control was observed to be worse in patients with long-term diabetes (HbA_1c_%, 9.0 ± 2.0 and 7.9 ± 2.1) than in those with new-onset disease. As expected, insulin secretion estimated with HOMA2-B was lower in the persons with long-term diabetes (%, 63.9 ± 45.9 and 81.1 ± 44.6) as compared to those with new-onset (Table 1). The prevalence of PDR was 5.5%, CKD was 26.7%, peripheral neuropathy was 85.6%, and non-fatal CVD was 14.9% (Table 1).

### Characteristics of Different Clusters in Patients With Adult Diabetes

**FIGURE 1:**
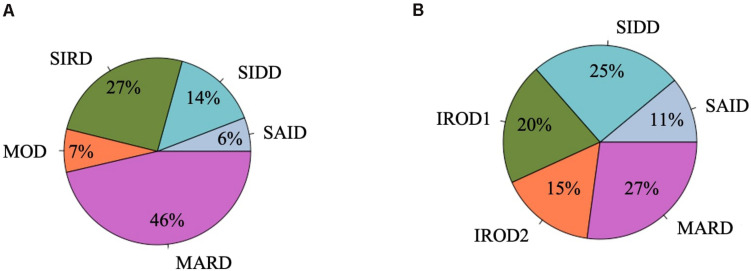
Frequency distribution of clusters in the DOLCE study. **(A)** Individuals with new-onset adult diabetes with less than 3 years of disease duration (*n* = 887). **(B)** Individuals with long-term adult diabetes with more than 3 years of disease duration (*n* = 1,253).

**FIGURE 2:**
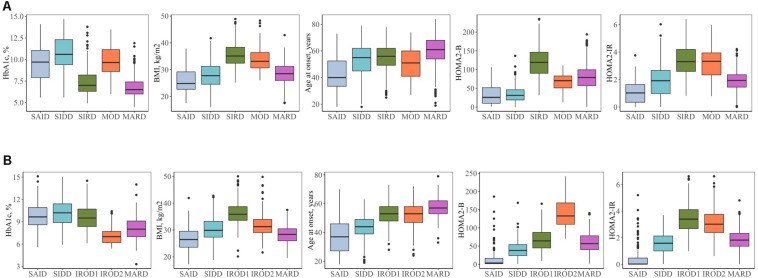
Clinical characteristics of the clusters. **(A)** Individuals with new-onset adult diabetes with less than 3 years of disease duration (*n* = 887). **(B)** Individuals with long-term adult diabetes with more than 3 years of disease duration (*n* = 1,253). SAID, severe autoimmune diabetes**;** SIDD, severe insulin-deficient diabetes; SIRD, severe insulin-resistant diabetes; MOD, mild obesity-related diabetes; MARD, mild age-related diabetes; IROD1 and 2, insulin-resistant obese diabetes 1 and 2; HOMA2-B, homoeostatic model assessment two estimates of β-cell function; HOMA2-IR, homoeostatic model assessment two estimates of insulin resistance.

Descriptive characteristics and frequency of the clusters in the patients with long-term and new-onset adult diabetes are presented in Figures 1, 2 and Supplementary Table 1.

Severe autoimmune diabetes (SAID) was twice prevalent in patients with long-term (11 and 6%) as compared to those with new-onset diabetes (Figure 1). Of these, in total 74 (37 and 43%) had clinical diagnosis as T2D. Individuals in this cluster had low BMI, younger age at diagnosis, and the lowest HOMA2-B and HOMA2-IR as compared to other clusters (Figure 2 and Supplementary Table 1). Individuals with SAID most frequently had insulin treatment, particularly among those with the long-term diabetes (84.1 and 58.8%), while oral antidiabetic treatment was more frequent in the new-onset group (9.4 and 27.5%) (Supplementary Table 1).

Severe insulin-deficient diabetes (SIDD) was almost twice as prevalent in the patients with long diabetes duration than in those with new-onset diabetes (25 and 14%) (Figure 1). This cluster was characterized by low insulin secretion as shown by HOMA2-B, relatively low BMI and the high HbA_1c_ than the other clusters (Figure 2 and Supplementary Table 1). The frequency of insulin therapy alone (42.6 and 25.6%) or in combination with oral anti glycemic medications (17.9 and 4.1%) was higher than in all other clusters apart from SAID for the patients with long-term and new-onset disease (Supplementary Table 1).

Severe insulin-resistant diabetes (SIRD) cluster occurred with 27% frequency in patients with new-onset diabetes (Figure 1). This cluster in the new-onset group was characterized by the highest insulin-resistance index HOMA2-IR (%, 3.4 ± 1.1) and HOMA2-B (%, 121.7 ± 42.2), elevated BMI and lower HbA_1c_ (Figure 2 and Supplementary Table 1). The insulin resistant obese patients with long-term diabetes differed from the original SIRD individuals. They exhibit similarly elevated HOMA2-IR (%, 3.5 ± 1.1) while insulin secretion index HOMA2-B was lower (%, 65.7 ± 27.8) and therefore we named this cluster insulin resistant obese diabetes 1 (IROD1) (Figure 2 and Supplementary Table 1). IROD1 cluster also showed the highest waist circumference among all clusters, reflecting the presence of an abdominal adiposity (Supplementary Table 1). Oral anti-glycemic treatment was common in long-term and new-onset groups (72.2 and 48.3%), however, long-term IROD1 reported more frequent use of sulfonylurea (61%). In addition, half of the individuals with new-onset diabetes (49.1%) and only 8.6% with long-term did not receive any anti-diabetic medications.

Mild obesity-related diabetes (MOD) cluster in new-onset group occurred with the frequency of 7% and was the smallest after SAID cluster (Figure 1). This group in the new-onset group was characterized by elevated BMI (kg/m^2^, 34.0 ± 4.9), HOMA2-IR (%, 3.3 ± 1.2), and moderately elevated HOMA2-B index (%, 66.9 ± 23.8). Patients with long-term diabetes in this cluster showed elevated BMI (kg/m^2^, 32.0 ± 4.5) and HOMA2-IR (%, 3.1 ± 1.1), but did not match their original MOD counterparts in the new-onset group by markedly elevated HOMA2-B index (%, 139.4 ± 38.8), and therefore we named them insulin resistant obese diabetes 2 (IROD2) (Figure 2 and Supplementary Table 1). Patients in both groups received often oral anti-hyperglycemic treatment (64.7 and 70.8%), or were controlled with diet and/or lifestyle intervention (27.9 and 23.6%) of long-term and new-onset diabetes, respectively (Supplementary Table 1).

Finally, mild age-related diabetes (MARD) was the largest cluster in both long-term and new-onset adult diabetes (27 and 46%) (Figure 1). This cluster was by definition characterized by the highest age at disease diagnosis (years, 59.7 ± 11.7 and 59.9 ± 11.7) and had similar characteristics across the two patients’ groups (Figure 2 and Supplementary Table 1).

### Risk of Diabetes Complications in Different Clusters

**FIGURE 3:**
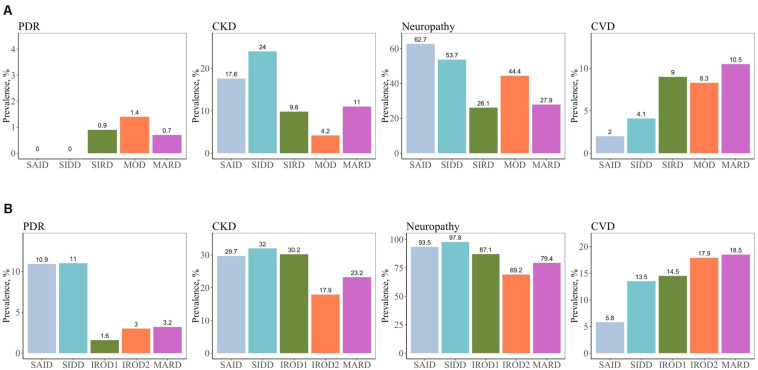
Prevalence of macro- and microvascular complications in different clusters of adult patients with the new-onset and long-term diabetes. **(A)** Individuals with new-onset adult diabetes with less than 3 years of disease duration (*n* = 887). **(B)** Individuals with long-term adult diabetes with more than 3 years of disease duration (*n* = 1,253). PDR, proliferative diabetic retinopathy; CKD, chronic kidney disease; CVD, cardiovascular disease.

We assessed the risk of macro- and microvascular complications of diabetes in each cluster using MARD as reference group, and adjusted for age, sex, and diabetes duration. In this cross-sectional study, patients with new-onset adult diabetes had few PDR events, and therefore analysis was not performed to calculate PDR risk in these patients. Only few cases (4.2%) had CKD in MOD cluster with the new onset diabetes (Figure 3 and Supplementary Table 1). Similar to the reference ANDIS study (Ahlqvist et al., 2018), in long-term group the SAID cluster was characterized with high prevalence of PDR (10.9%) with OR of 9.32-fold (95% CI, 2.15–40.46, *p* = 0.003) relative to MARD cluster. Prevalence of CVD in this cluster was detected to be lower than in the other clusters (5.8%). The SIDD cluster had similarly to SAID high prevalence and increased risk of PDR (11%, OR 2.42, 95% CI, 0.96– 6.07, *p* = 0.06) relative to MARD cluster (Figure 3, Table 2, and Supplementary Table 1). Prevalence of CKD was found to be elevated in all severe clusters, i.e., SAID (29.7%), SIDD (32%), and insulin-resistant obese diabetes 1 (IROD1) (30.2%) conferring an increased risk of 2.59-fold (1.34– 5.00, *p* = 0.005), 2.03-fold (1.21– 3.40, *p* = 7.1 × 10^–3^), and 1.63-fold (1.08 –2.46, *p* = 0.02) relative to MARD in patients with long diabetes duration. In contrast to ANDIS, neuropathy prevalence was high across all clusters, but the relative risk was highest in SIDD cluster (OR 13.60, 95% CI 5.20–35.50, *p* = 1.10 × 10^–7^). In general, the IROD2 cluster exhibited the lowest prevalence of all microvascular complications, particularly CKD and neuropathy.

**TABLE 2:** Risk of macro- and microvascular complications in different clusters in long-term adult diabetes relative to MARD.

Complications	SAID		SIDD		IROD1		IROD2	
	OR (95% CI)	*P*-value	OR (95% CI)	*P*-value	OR (95% CI)	*P*-value	OR (95% CI)	*P*-value
PDR	9.32 (2.15, 40.46)	0.003*	2.42 (0.96, 6.07)	0.06	0.53 (0.16, 1.79)	0.31	0.73 (0.23, 2.32)	0.59
CKD	2.59 (1.34, 5.00)	0.005*	2.03 (1.21, 3.40)	7.1 × 10^–3^*	1.63 (1.08, 2.46)	0.02*	0.85 (0.53, 1.39)	0.52
Neuropathy	3.30 (1.29, 8.43)	0.01*	13.60 (5.20, 35.50)	1.0 × 10^–7^*	2.08 (1.28, 3.37)	3.0 × 10^–3^*	0.70 (0.45, 1.09)	0.12
CVD	0.61 (0.25, 1.51)	0.3	1.13 (0.60, 2.11)	0.71	1.11 (0.68, 1.8)	0.68	1.44 (0.87, 2.36)	0.15

PDR, proliferative diabetic retinopathy; CKD, chronic kidney disease; CVD, cardiovascular disease; OR, Odds Ratios; 95% CI, 95% confidence intervals; and p-values were calculated using logistic regression adjusted for sex, age, and diabetes duration. *Significant after adjustment for the multiple testing using FDR (<0.05).

### Genetic Analyses

We analyzed variants reported in the DIAGRAM GWAS meta-analyses (Morris et al., 2012) to provide insights on the key T2D SNPs associated with protective phenotype of insulin resistant obese (IROD2) cluster in patients with long-term diabetes relative to all other clusters (excluding SAID due to the lack of genetic information in this cluster). The top SNP was rs7903146 in *TCF7L2* (OR, 95%CI; 0.54, 0.39–0.74, *p* = 0.0001), which showed significantly lower frequency of the risk T-allele in IROD2 than in IROD1 (*p* = 0.008), SIDD (*p* = 0.001) and MARD (*p* = 0.0003) (Table 3). The same directionality for lower risk allele frequencies in IROD2 was also found for *KCNQ1* locus (rs163184, 0.67, 0.52–0.88, *p* = 0.003), *NOTCH2* locus (rs1493694, 0.49, 0.30– 0.80, *p* = 0.004), locus in the gene related to cell-matrix interplay *ADAMTS9* (rs6795735: 0.72, 0.56– 0.93, *p* = 0.012), loci in genes *ZFAND6* (rs11634397: 0.73, 0.56– 0.94, *p* = 0.016), and *ZFAND3* (rs4299828: 0.73, 0.54– 0.99, *p* = 0.042). On the contrary, higher frequencies were observed for the risk alleles of SNPs in the gene related to cell signaling *PTPRD* (rs16927668: 1.40, 1.03–1.91, *p* = 0.033). However, after adjustment for multiple testing using Bonferroni correction, only variant rs7903146 in *TCF7L2* remained statistically significant (*p*-value _*IROD*__2__vs. all clusters_ = 0.008, *p*-value _*IROD*__2__vs. SIDD_ = 0.048, *p*-value _*IROD*__2__vs. MARD_ = 0.002) (Table 3).

**TABLE 3:** Top T2D SNPs nominally associated with IROD2 cluster in individuals with long-term diabetes.

SNP	Gene	Chr	BP	Risk allele	RAF	IROD2 vs. all clusters		IROD2 vs. IROD1		IROD2 vs. SIDD		IROD2 vs. MARD	
						OR (95% CI)	*P*-value	OR (95% CI)	*P*-value	OR (95% CI)	*P*-value	OR (95% CI)	*P*-value
rs7903146	*TCF7L2*	10	114758349	T	0.28	0.54 (0.39, 0.74)	0.0001*	0.61 (0.42, 0.88)	0.008	0.54 (0.37, 0.77)	0.001*	0.45 (0.31, 0.65)	0.0003*
rs163184	*KCNQ1*	11	2847069	G	0.50	0.67 (0.52, 0.88)	0.003	0.60 (0.44, 0.82)	0.001	0.74 (0.54, 1.01)	0.059	0.71 (0.52, 0.98)	0.036
rs10923931	*NOTCH2*	1	120517959	T	0.13	0.49 (0.30, 0.80)	0.004	0.52 (0.29, 0.90)	0.020	0.50 (0.29, 0.87)	0.014	0.45 (0.26, 0.78)	0.004
rs6795735	*ADAMTS9*	3	64705365	C	0.59	0.72 (0.56, 0.93)	0.012	0.68 (0.50, 0.93)	0.015	0.78 (0.58, 1.06)	0.113	0.72 (0.53, 0.97)	0.033
rs11634397	*ZFAND6*	15	80432222	G	0.66	0.73 (0.56, 0.94)	0.016	0.71 (0.51, 0.99)	0.041	0.70 (0.51, 0.96)	0.025	0.76 (0.56, 1.03)	0.081
rs16927668	*PTPRD*	9	8369533	T	0.18	1.40 (1.03, 1.91)	0.033	1.45 (1.00, 2.10)	0.047	1.53 (1.04, 2.24)	0.032	1.25 (0.85, 1.82)	0.255
rs4299828	*ZFAND3*	6	38177667	A	0.81	0.73 (0.54, 0.99)	0.042	0.65 (0.46, 0.93)	0.018	0.80 (0.55, 1.15)	0.227	0.79 (0.55, 1.13)	0.195
rs459193	*ANKRD55*	5	55806751	G	0.72	1.36 (1.01, 1.84)	0.044	1.30 (0.90, 1.86)	0.159	1.32 (0.92, 1.88)	0.128	1.44 (1.01, 2.07)	0.045

Association of T2D SNPs with IROD2 cluster as case group. OR and 95% CI were calculated using logistic regression adjusted for sex and age. Chr, chromosome; BP, base pair; RAF, risk allele frequency. Number of individuals in the analyses IROD2 vs. all clusters: n = 837, IROD2 vs. IROD1: n = 357, IROD2 vs. SIDD: n = 355, IROD2 vs. MARD: n = 415. *Significant after adjustment for the multiple testing using Bonferroni correction (p < 0.05).

## Discussion

The findings from this observational study demonstrate that the novel approach using SAID, SIDD, SIRD, MOD, and MARD clustering of diabetes subgroups (Ahlqvist et al., 2018) is replicated in the patients with adult diabetes from northern Ukraine. In accordance with the published studies, the SIDD cluster had the highest prevalence of retinopathy and neuropathy, and the insulin resistant subgroups were linked to high risk of CKD. In contrast to Scandinavian and German populations (Ahlqvist et al., 2018; Zaharia et al., 2019) and similar to the insulin-deficient insulin-resistant subgroup in a recently published Asian-Indian cohort (Anjana et al., 2020), SIDD cluster in this cohort also showed high risk of CKD. With longer duration of diabetes, the clusters might change, and insulin resistant obese cases could be challenging to match to the original SIRD and MOD cluster of corresponding new-onset diabetes. In general, patients with long-term diabetes and preserved β-cell function demonstrated better glycemic control measured by HbA_1c_ and lower risk of all microvascular complications than expected.

An important observation was lower insulin secretion in patients from northern Ukraine with new-onset adult diabetes compared to the Swedish ANDIS cohort. The history of Ukraine during the first half of the 20^*th*^ century could contribute to this difference. It has been previously reported that children born to parents exposed to the Ukrainian Holodomor famine (1932–1933) showed increased risk of developing T2D later in life (Lumey et al., 2015). Early life exposure to starvation might exhibit detrimental effects, which might result in malformation of the pancreas, thereby reducing the number of β-cells (β-cell mass) and/or affecting the secretory activity of β-cells (β-cell function) (Chen et al., 2017). The decrease in insulin output as a consequence of the reduced β-cell mass could be caused by a progressive β-cell death through established starvation-induced processes on apoptosis and disrupted autophagy (Marrif and Al-Sunousi, 2016). Thus, intrauterine programming may be related to the restricted insulin secretory capacity of pancreatic islets as a response to the demands imposed by increased insulin resistance linked to obesity in adult life. This could contribute to more severe manifestation of diabetes in the Ukrainian population. Lower frequency of insulin therapy in Ukrainian patients with new-onset diabetes in adults despite more profound insulin secretory defects compared to the ANDIS cohort could indicate that many more patients might benefit from early intensified treatments targeting preservation of insulin secretion. Naturally, discovering new ways to maintain or refine β-cell mass via enhancing β-cell survival and/or reducing apoptosis is of specific interest to investigate.

SIDD was the most severe cluster in this study, which supported consistently documented high prevalence of retinopathy across all published studies, and also showed elevated prevalence of CKD. These results are opposite to ANDIS and other cohorts (Ahlqvist et al., 2018; Zaharia et al., 2019; Zou et al., 2019) showing increased risk of CKD conferred to SIRD cluster, but somewhat similar to the recently reported findings in Asian-Indian population (Anjana et al., 2020) demonstrating increased risk of CKD in the combined insulin-deficient insulin-resistant cluster. One potential explanation for this observation could reflect and support the above-mentioned effects and malformations of organs as a consequence of abnormal intrauterine programming, which may lead to increased susceptibility to vascular complications in patients with adult diabetes later in life. In support of this, studies from the Dutch famine birth cohort have reported increased risk of microalbuminuria in adults after prenatal exposure to famine (Painter et al., 2005). Researchers suggested that fetal undernutrition may lead to lower nephron endowment, reduced number of glomeruli and consequently hyperfiltration, which may cause glomerular damage and lead to a reduction in renal function.

In line with study by Zaharia et.al of German patients with adult diabetes (Zaharia et al., 2019), the highest waist circumference was observed in the IROD1 cluster of long-term diabetes. From this perspective, the data suggest that IROD1 is associated with abdominal (visceral) type of adiposity as compared to IROD2 in which subcutaneous adipose tissue is prevailing fat deposition. Abdominal adiposity is considered to be much unhealthier than subcutaneous fat accumulation (Kahn et al., 2001) and associated with increased insulin resistance, inflammation, atherosclerosis, and vascular complications (Reijrink et al., 2019) as well as increased mortality (Christakoudi et al., 2020). Similarly, in the German Diabetes Study, the patients with newly diagnosed adult diabetes in the SIRD cluster demonstrated the highest hepatocellular lipid content and the highest prevalence of hepatic fibrosis at 5-year follow-up (Zaharia et al., 2019). This supports the idea of visceral obesity contributing to elevated risks of metabolic disorders and vascular complications in SIRD.

An interesting finding of the clustering approach in long-term adult diabetes was related to the IROD2 cluster with preserved β-function. It is tempting to speculate that people in the IROD2 cluster could have escaped starvation during the historical famines and thereby have had their β-cell function preserved relative to the people who experienced undernutrition during times of exposure. However, we do not have information on caloric intake during famine periods; hence, this hypothesis could only be tested in a population where such information exists (e.g., Dutch famine birth cohort).

To shed light on possible underlying genetic factors of associated with beta-cell function in IROD2 cluster, we compared the frequency of established T2D variants (Morris et al., 2012). The genetic results identified consistently lower frequency of risk alleles at the *TCF7L2* locus in IROD2 cluster compared to each other cluster in this group of patients. Since 2007, polymorphisms of *TCF7L2*, encoding for transcription factor-7–like 2, are considered to be guilty of β-cells dysfunction and increased risk of diabetes in different ethnic populations (Scott et al., 2007; Dimas et al., 2014). *TCF7L2* is a member of the T-cell–specific high-mobility group box-containing family of transcription factors, that acts through Wnt-β-catenin dependent and independent pathways (Karve et al., 2020) and coordinates expression of various genes regulating cell cycle and fate determination. In pancreas *TCF7L2* was shown to play a crucial role in regulation of β-cell survival and proliferation rate. Moreover, *TCF7L2* controls the expression of genes involved in insulin granule fusion at the plasma membrane through syntaxin repression, affecting insulin secretion levels (da Silva Xavier et al., 2009). According to our results, *TCF7L2* rs7903146, which is a lead among T2D susceptibility loci (Hattersley, 2007), associated with more severe T2D phenotypes, and the lower frequencies of the risk allele appear to be associated with the protection against progression toward several vascular complications. As rs7903146 has been shown to reside in islet-selective open chromatin (Gaulton et al., 2010), this clearly motivates further metabolic studies of this group to identify epigenetic factors that play multifaced roles.

Second top signal in IROD2 group of long-term diabetes suggested reduced frequency of the risk allele in the imprinting gene *KCNQ1* (rs 163,184) that has also been shown to be expressed in the pancreatic β-cells and to act through impaired islet function on the risk of future T2D (Jonsson et al., 2009). *KCNQ1* locus was first discovered as a top signal in the two GWAS for T2D from Japan (Unoki et al., 2008; Yasuda et al., 2008) and identified as a GWAS locus for parent-of-origin effects in a large family-based study from Iceland (Kong et al., 2009). Functional and analyses of imprinting status of this genomic region suggested that metabolic effects conferred by the risk alleles at the *KCNQ1* locus target the cyclin-dependent kinase inhibitor *CDKN1C* playing a key role in regulating pancreatic β-cells proliferation and development (Kassem et al., 2001). Expression of both *KCNQ1* and *CDKN1C* demonstrated to exhibit temporal effects in fetal and adult human pancreas and islets emphasizing that the diabetes risk may be mediated in early development (Travers et al., 2013). In line with this notion, unbalanced placental expression of *CDKN1C* has been associated with intrauterine growth retardation (McMinn et al., 2006). This further supports the idea of possible contributing role of intrauterine programming of reduced pancreatic β-cell function in this population.

Several other genetic loci might deserve attention such as consistently lower frequency of risk variants in *NOTCH2* rs1493694 in IROD2 cluster. *NOTCH2*, which encodes for neurogenic locus notch homolog protein 2, is known to be responsible for regulating interactions between adjacent cells. It was demonstrated that *NOTCH2* is involved in insulin secretion and sensitivity as well as growth and development of the pancreas (Jonsson et al., 2013). The protein’s extracellular domain consists of multiple EGF-like repeats while intracellular domain is involved in cell signaling affecting a variety of developmental processes controlling cell fate determination. Altered *NOTCH2* expression was found to be related to diabetic complications (Rasheed et al., 2017).

It is worth discussing that comparison of cluster characteristics in patients with longer duration of diabetes demonstrated that we for sure could not tell which of the two insulin resistant obese clusters in long-term would match the original SIRD and MOD. The original SIRD cluster in the new-onset group was characterized by high HOMA2-IR and HOMA2-B. In long-term diabetes, we could not observe the original phenomenon of simultaneously elevated both indices. To avoid confusion with the original clusters, we named the groups IROD1 in which for the given high HOMA2-IR a reduced HOMA2-B was observed, and IROD2 in which for the relatively lower HOMA2-IR higher HOMA2-B was observed. One possible explanation for the long-term HOMA-B changes in IROD1 group could be related to more frequent use of sulfonylurea drugs (61 vs. 48%, *p* = 0.007), which increase insulin secretion in short term but are considered to lead to lower insulin secretion in long term (Maedler et al., 2005; Shin et al., 2012). Additionally, these clusters differed in respect to the risk of complications with IROD2 having lowest prevalence of CKD. The IROD2 cluster was characterized by better insulin secretion and reduced frequency of the risk allele in the *TCF7L2* gene in line with genetic data in the SIRD cluster from ANDIS (Aly et al., 2020). These findings emphasize that in long-term cases changes in HOMA2-IR and HOMA2-B might occur and rather a general fit to the cluster might be considered as opposed to the given preference to one of these measures, and genetic information could be beneficial to assign people to the original SIRD cluster.

### Limitations

The analyses in the present study were conducted on the patients with adult diabetes from a defined population of northern Ukraine (Chernihiv and Kyiv regions) with a history of Holodomor famine. Therefore, the findings might not be generalized to the other regions of Ukraine. The cohort comprised of adult patients with established diabetes, and the blood sampling was conducted at the study visit instead of the time of diagnosis, which limits the prognostic assessment complications risk in this cross-sectional study. Follow-up data would be required to determine if the difference between the SIRD and MOD groups in new onset diabetes and the IROD1 and IROD2 groups are due to phenotype changes over time, and in that case which of the two indices of insulin resistance and β-cell function (HOMA2-IR or HOMA2-B) in patients with long-term would have a better fit to the original insulin resistant SIRD and MOD clusters coined for ANDIS. An alternative explanation is poor performance of the clustering algorithm due to the relatively small cohort size. It has been seen also in ANDIS that the MOD and SIRD clusters are the least stable but that a larger sample size can improve stability and reproducibility of the clusters. There were more women than men in this cohort, which could potentially give gender-specific effects. However, all analyses were separately performed in men and women, and gave similar results. The K_*mean*_ clustering algorithm used in the current approach presumes that all the clustering variables have the same weight. Nevertheless, giving variables different weights or prioritizing importance or using another clustering algorithm might improve the approach. All patients were recruited at the primary health care centers or outpatient clinic, which minimized the bias related to recruitment of severe diabetes patients admitted to the hospital or being on ward. Although the sample size is limited for genetic analyses, the power calculations showed that significant effects (*p* < 0.00014) would be reached for variants with the Genotype Relative Risk (disease probability for individuals with 1 risk allele divided by disease probability for individuals with 0 risk alleles) above 1,8 (Skol et al., 2006; Goncalo Abecasis et al., 2017).

## Conclusion

In conclusion, pathophysiology-based clustering is undoubtedly beneficial for diagnosing different subtypes of adult diabetes related to risk of micro- and macrovascular complications. Assessment of GADA is prerequisite to correctly re-classify SAID patients with adult diabetes, which in the clinical practice can be misclassified as T2D. It can be a clear advantage for the patients belonging to the SIDD cluster to start treatment with insulin or other therapeutic modalities at an earlier stage in order to preserve and maintain β-cell function. The persons with long-term diabetes assigned to IROD2 cluster exhibited preserved insulin secretion and lower risk for microvascular complications. Thus, this cluster represents an interesting subgroup of patients for further investigations of protective mechanisms. The current diabetes cluster approach could be further refined and optimized by including other new biomarkers derived from ongoing omics studies.

## Data Availability Statement

The datasets presented in this article are not readily available because of GDPR and ethical restrictions. Requests to access the datasets should be directed to valeriya.lyssenko@uib.no.

## Ethics Statement

The studies involving human participants were reviewed and approved by the Ethics Committee at “Regional Hospital,” Chernihiv, Ukraine (approval number Dnr17/2011-09-14) and Regional Committee for Medical and Health Research (Ethics, South-east, Panel A, approval number 2019/28968). The patients/participants provided their written informed consent to participate in this study.

## Author Contributions

OF did the statistical analyses, data interpretation, and wrote the manuscript. TO, EA, and OA assisted in statistical analyses. LC, NK, TS, and TB performed recruitment of patients, data acquisition, and clinical interpretation. OS, TO, EA, LG, and PN contributed to editing of the manuscript and data interpretation. VL conceived and designed the study, planned the analyses, supervised all parts of the study, interpreted the data, and wrote the manuscript. All authors contributed to the interpretation of the data, and approved the final version of the manuscript.

## Conflict of Interest

The authors declare that the research was conducted in the absence of any commercial or financial relationships that could be construed as a potential conflict of interest.
