# Supplementary material for: Novel Reclassification of Adult Diabetes Is Useful to Distinguish Stages of β-Cell Function Linked to the Risk of Vascular Complications: The DOLCE Study From Northern Ukraine
Source: Front Genet. 2021 Jul 2;12:637945. doi: 10.3389/fgene.2021.637945 (PMC8283002; doi:10.3389/fgene.2021.637945)
Supplement: Supplementary file 1 [file Data_Sheet_1.pdf]

frontiers

Supplementary Material

Table of Contents

1 Supplemental Figures *2*

1.1 Supplemental Figure 1: Flowchart of the data preparation (QC) for cluster analysis in GADA negative individuals with adult diabetes. 2

1.2 Supplemental Figure 2. Visualization of principal component analysis. 3

2 Supplemental tables *4*

2.1 Supplemental Table 1: Clinical characteristics and prevalence of macro- and microvascular complications in different clusters in the DOLCE cohort. 4

A. New-onset adult diabetes (n= 887). 4

B. Long-term adult diabetes (n = 1,253). 6

2.2. Supplemental Table 2: Association of T2D SNPs with IROD2 cluster in individuals with long-term adult diabetes. 8

2.2 Supplemental Table 3: Clinical characteristics and prevalence of macro- and microvascular complications in the entire DOLCE cohort. 13

2.3 Supplemental Table 4. Risk of macro- and microvascular complications in new-onset adult diabetes group in different clusters relative to MARD cluster (n=887). 15

1 Supplemental Figures

1.1 Supplemental Figure 1: Flowchart of the data preparation (QC) for cluster analysis in GADA negative individuals with adult diabetes.

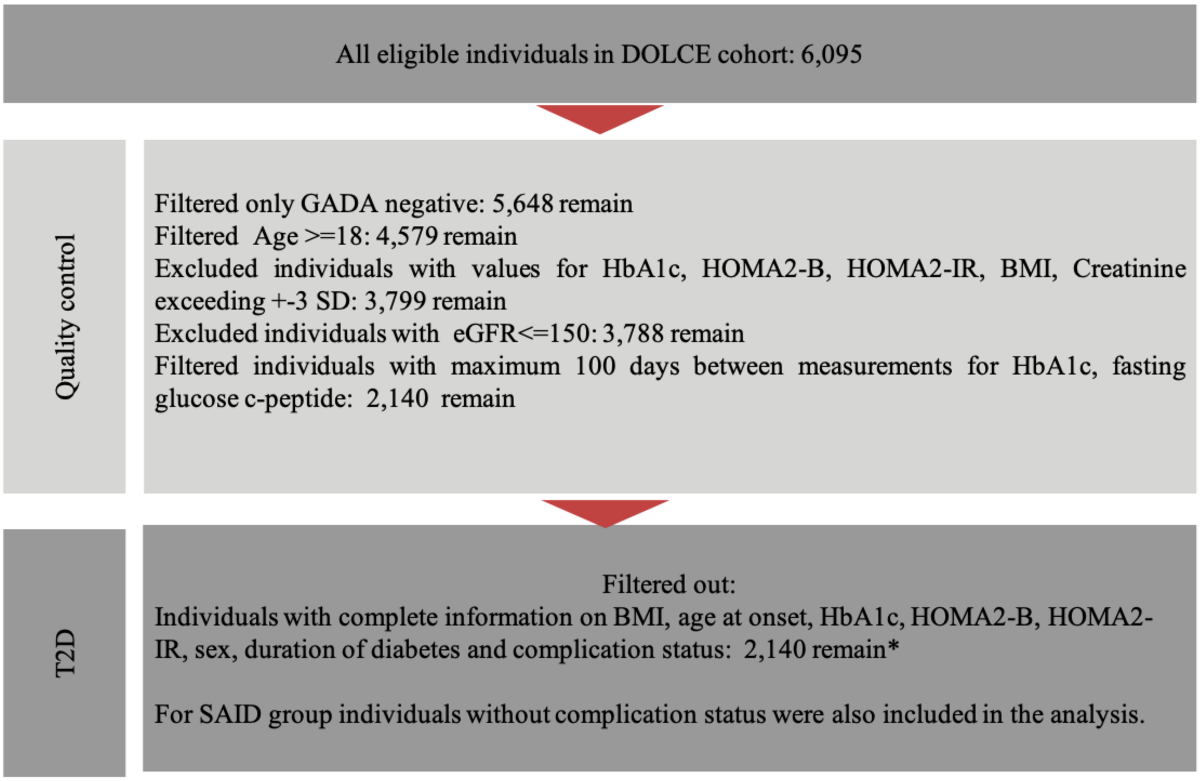

1.2 Supplemental Figure 2. Visualization of principal component analysis.

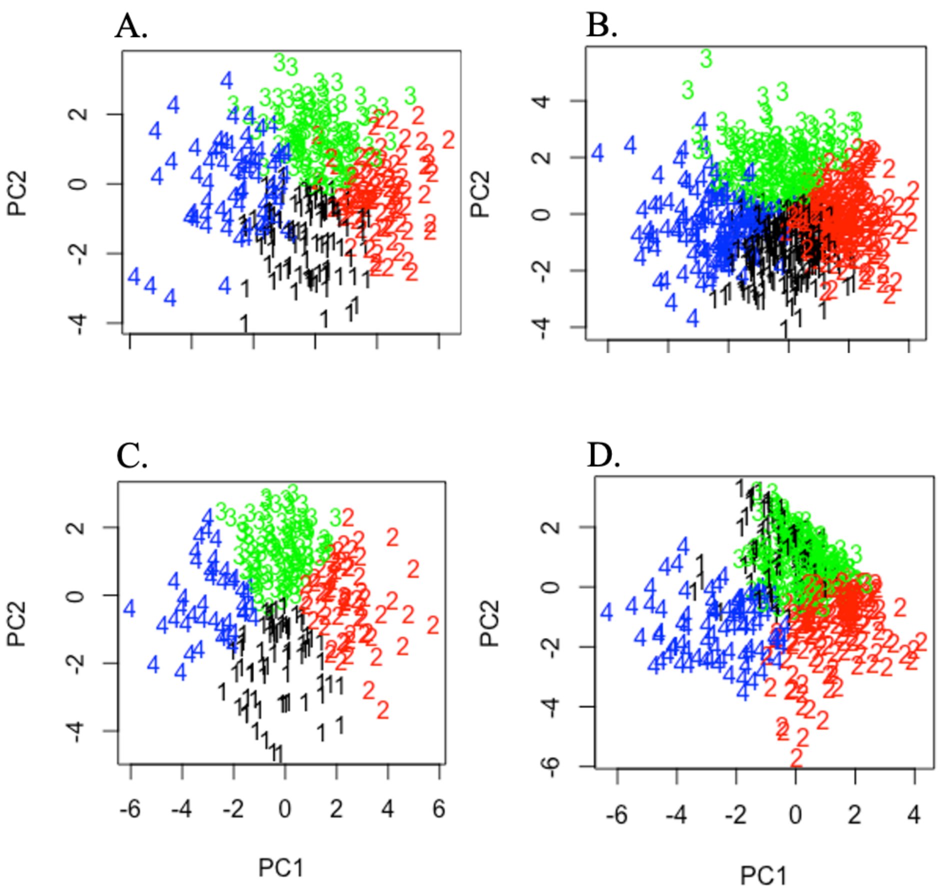

A, B - long-term adult diabetes group (A – men, B – women), duration of diabetes > 3 years; C, D - new-onset adult diabetes group, duration of diabetes <= 3 years (C – men, D – women). MOD/IROD2 – 1, MARD – 2, SIDD – 3, SIRD/IROD1 – 4.

2 Supplemental tables

2.1 Supplemental Table 1: Clinical characteristics and prevalence of macro- and microvascular complications in different clusters in the DOLCE cohort.

A. New-onset adult diabetes (n= 887).

Phenotype	SAID	SIDD	SIRD	MOD	MARD
N, (men, %)	51 (57%)	121 (30%)	234 (31%)	72 (100%)	409 (28%)
Age at visit, years	44 (14.7)	54 (13.2)	55.8 (9.8)	51.7 (11.7)	60.9 (11.7)
Age at onset of diabetes, years	42.6 (14.5)	52.6 (13.2)	54.8 (9.8)	50.7 (11.7)	59.9 (11.7)
Diabetes duration, years	1.2 (1.2)	1.5 (1.3)	0.9 (1.1)	1 (1.2)	1.1 (1.1)
HbA1c, %	9.7 (2.2)	10.7 (2.1)	7.4 (1.6)	9.8 (1.8)	6.8 (1.1)
HbA1c, mmol/mol	82 (24.2)	93.8 (22.9)	57.8 (17)	84.1 (20.2)	50.6 (12.5)
BMI, kg/m2	25.8 (4.1)	27.9 (5.3)	35.6 (4.8)	34 (4.9)	28.7 (4.1)
Waist, cm	88.1 (11.6)	92.1 (12.8)	107.1 (12.5)	110.7 (12.8)	92.1 (11.4)
HOMA2-B	36.5 (30.9)	36.4 (23.1)	121.7 (42.2)	66.9 (23.8)	79.2 (33.3)
HOMA2-IR	1.1 (1)	2 (1.2)	3.4 (1.1)	3.3 (1.2)	1.9 (0.7)
C-peptide, nmol/l	0.4 (0.4)	0.7 (0.4)	1.4 (0.4)	1.2 (0.4)	0.8 (0.3)
Without treatment, %	9.8%	20.7%	49.1%	23.6%	62.1%
Tablets, %	27.5%	49.6%	48.3%	70.8%	34.2%
Insulin, %	58.8%	25.6%	1.7%	1.4%	2.9%
Tablets and insulin, %	3.9%	4.1%	0.9%	4.2%	0.7%
Sulfonylurea, %	27%	36%	25%	47%	21%

PDR, %	-	-	0.9%	1.4%	0.7%
CKD, %	17.6%	24%	9.8%	4.2%	11%
Neuropathy, %	62.7%	53.7%	26.1%	44.4%	27.9%
CVD, %	2%	4.1%	9%	8.3%	10.5%

B. Long-term adult diabetes (n = 1,253).

Phenotype	SAID	SIDD	IROD1	IROD2	MARD
N, (men, %)	138 (44%)	319 (29%)	255 (35%)	201 (31%)	340 (36%)
Age at visit, years	51 (13.8)	58 (8.3)	62.1 (7.9)	61.7 (8.4)	67.2 (7.3)
Age at onset of diabetes, years	38.1 (13.2)	43.4 (7.7)	53 (8.1)	52.5 (8.7)	57.6 (6.8)
Diabetes duration, years	12.7 (8.3)	14.5 (8.3)	9 (5.1)	9.2 (5.6)	9.5 (5.3)
HbA1c, %	9.7 (1.8)	10.2 (1.8)	9.6 (1.7)	7.1 (1.1)	8.1 (1.5)
HbA1c, mmol/mol	82.7 (19.8)	87.8 (19.5)	81.5 (18.6)	54.2 (11.9)	64.9 (16)
BMI, kg/m2	26.8 (4.4)	30.3 (4.5)	36.4 (4.4)	32 (4.5)	28.3 (3.3)
Waist, cm	89.8 (13.1)	98.3 (11.8)	109.8 (10.2)	100.5 (11.1)	94.6 (10)
HOMA2-B	15.6 (28.1)	40.2 (22.3)	65.7 (27.8)	139.4 (38.8)	59.7 (25.6)
HOMA2-IR	0.5 (0.9)	1.6 (0.8)	3.5 (1.1)	3.1 (1.1)	1.9 (0.8)
C-peptide, nmol/l	0.2 (0.3)	0.6 (0.3)	1.2 (0.4)	1.3 (0.4)	0.7 (0.3)
Without treatment, %	2.9%	4.1%	8.6%	27.9%	16.8%
Tablets, %	9.4%	35.4%	72.2%	64.7%	60.9%
Insulin, %	84.1%	42.6%	8.6%	6%	17.9%
Tablets and insulin, %	3.6%	17.9%	10.6%	1.5%	4.4%
Sulfonylurea, %	9%	37%	61%	48%	54%
PDR, %	10.9%	11%	1.6%	3%	3.2%
CKD, %	29.7%	32%	30.2%	17.9%	23.2%
Neuropathy, %	93.5%	97.8%	87.1%	69.2%	79.4%

CVD, %	5.8%	13.5%	14.5%	17.9%	18.5%

SAID **-** severe autoimmune diabetes**,** SIDD – severe insulin-deficient diabetes, SIRD – severe insulin-resistant diabetes, MOD – mild obesity-related diabetes, MARD – mild age-related diabetes, IROD1 and 2 – insulin-resistant obese diabetes 1 and 2. HOMA2-B=homoeostatic model assessment 2 estimates of β-cell function. HOMA2-IR=homoeostatic model assessment 2 estimates of insulin resistance. Data are mean ± (SD). PDR – proliferative diabetic retinopathy. CKD – chronic kidney disease. CVD - cardiovascular disease. *Data for PDR in individuals from SAID cluster was available for 92 individuals from the group with long-term adult individuals.

frontiers

2.2. Supplemental Table 2: Association of T2D SNPs with IROD2 cluster in individuals with long-term adult diabetes.

SNP	Gene	Chr	BP	Risk allele	RAF	IROD2 vs. all clusters		IROD2 vs. IROD1		IROD2 vs. SIDD		IROD2 vs. MARD	
						OR (95% CI)	P-value	OR (95% CI)	P-value	OR (95% CI)	P-value	OR (95% CI)	P-value
rs7903146	*TCF7L2*	10	114758349	T	0.28	0.54 (0.39, 0.74)	0.0001*	0.61 (0.42, 0.88)	0.008	0.54 (0.37, 0.77)	0.001*	0.45 (0.31, 0.65)	0.0003*
rs163184	*KCNQ1*	11	2847069	G	0.50	0.67 (0.52, 0.88)	0.003	0.60 (0.44, 0.82)	0.001	0.74 (0.54, 1.01)	0.059	0.71 (0.52, 0.98)	0.036
rs10923931	*NOTCH2*	1	120517959	T	0.13	0.49 (0.30, 0.80)	0.004	0.52 (0.29, 0.90)	0.020	0.50 (0.29, 0.87)	0.014	0.45 (0.26, 0.78)	0.004
rs6795735	*ADAMTS9*	3	64705365	C	0.59	0.72 (0.56, 0.93)	0.012	0.68 (0.50, 0.93)	0.015	0.78 (0.58, 1.06)	0.113	0.72 (0.53, 0.97)	0.033
rs11634397	*ZFAND6*	15	80432222	G	0.66	0.73 (0.56, 0.94)	0.016	0.71 (0.51, 0.99)	0.041	0.70 (0.51, 0.96)	0.025	0.76 (0.56, 1.03)	0.081
rs16927668	*PTPRD*	9	8369533	T	0.18	1.40 (1.03, 1.91)	0.033	1.45 (1.00, 2.10)	0.047	1.53 (1.04, 2.24)	0.032	1.25 (0.85, 1.82)	0.255
rs4299828	*ZFAND3*	6	38177667	A	0.81	0.73 (0.54, 0.99)	0.042	0.65 (0.46, 0.93)	0.018	0.80 (0.55, 1.15)	0.227	0.79 (0.55, 1.13)	0.195
rs459193	*ANKRD55*	5	55806751	G	0.72	1.36 (1.01, 1.84)	0.044	1.30 (0.90, 1.86)	0.159	1.32 (0.92, 1.88)	0.128	1.44 (1.01, 2.07)	0.045
rs13233731	*KLF14*	7	130437689	G	0.55	1.27 (0.98, 1.64)	0.073	1.32 (0.97, 1.79)	0.074	1.30 (0.95, 1.79)	0.097	1.25 (0.92, 1.69)	0.153
rs8182584	*PEPD*	19	33909710	T	0.41	0.79 (0.61, 1.03)	0.084	0.84 (0.62, 1.15)	0.279	0.75 (0.55, 1.03)	0.075	0.84 (0.62, 1.15)	0.279
rs516946	*ANK1*	8	41519248	C	0.78	0.76 (0.56, 1.04)	0.086	0.78 (0.54, 1.13)	0.184	0.81 (0.56, 1.18)	0.267	0.66 (0.45, 0.98)	0.037
rs10203174	*THADA*	2	43690030	C	0.90	1.54 (0.93, 2.55)	0.090	1.79 (1.02, 3.15)	0.044	1.24 (0.68, 2.25)	0.481	1.66 (0.93, 2.97)	0.087
rs780094	*GCKR*	2	27741237	C	0.58	1.20 (0.93, 1.55)	0.161	1.26 (0.94, 1.68)	0.125	1.17 (0.86, 1.60)	0.326	1.20 (0.88, 1.64)	0.248

rs7569522	*RBMS1*	2	161346447	A	0.45	0.84 (0.64, 1.08)	0.176	0.85 (0.63, 1.15)	0.287	0.81 (0.58, 1.12)	0.202	0.81 (0.59, 1.10)	0.176
rs12899811	*PRC1*	15	91544076	G	0.35	0.83 (0.63, 1.09)	0.181	0.72 (0.53, 0.98)	0.039	1.06 (0.78, 1.44)	0.725	0.81 (0.59, 1.12)	0.200
rs10842994	*KLHDC5*	12	27965150	C	0.76	1.23 (0.90, 1.66)	0.191	1.13 (0.79, 1.60)	0.502	1.45 (1.02, 2.06)	0.039	1.30 (0.90, 1.87)	0.159
rs12497268	*PSMD6*	3	64090363	G	0.82	0.81 (0.59, 1.12)	0.198	0.70 (0.47, 1.04)	0.081	0.88 (0.60, 1.28)	0.502	0.79 (0.54, 1.16)	0.230
rs3104413	*HLA*	6	32582650	G	0.12	0.75 (0.49, 1.17)	0.202	0.82 (0.50, 1.35)	0.443	0.81 (0.49, 1.34)	0.420	0.62 (0.38, 1.04)	0.069
rs2334499	*DUSP8*	11	1696849	T	0.40	0.85 (0.65, 1.10)	0.212	0.83 (0.61, 1.13)	0.235	0.86 (0.63, 1.18)	0.349	0.80 (0.58, 1.09)	0.159
rs11257655	*CDC123/CAMK1D*	10	12307894	T	0.20	0.81 (0.58, 1.14)	0.231	0.96 (0.64, 1.44)	0.834	0.68 (0.45, 1.01)	0.057	0.86 (0.59, 1.27)	0.460
rs10278336	*GCK*	7	44245363	A	0.59	1.16 (0.89, 1.50)	0.266	1.11 (0.82, 1.50)	0.514	1.22 (0.89, 1.67)	0.223	1.11 (0.82, 1.51)	0.479
rs4402960	*IGF2BP2*	3	185511687	T	0.33	0.86 (0.65, 1.13)	0.269	0.80 (0.58, 1.10)	0.162	1.04 (0.75, 1.46)	0.796	0.80 (0.58, 1.10)	0.176
rs243088	*BCL11A*	2	60568745	T	0.46	0.89 (0.69, 1.15)	0.371	0.97 (0.72, 1.30)	0.819	0.91 (0.67, 1.24)	0.567	0.83 (0.61, 1.12)	0.219
rs2261181	*HMGA2*	12	66212318	T	0.09	1.21 (0.79, 1.84)	0.376	1.14 (0.68, 1.91)	0.630	1.05 (0.63, 1.73)	0.861	1.64 (0.96, 2.80)	0.070
rs4502156	*C2CD4A*	15	62383155	T	0.59	1.12 (0.87, 1.45)	0.378	0.93 (0.68, 1.27)	0.638	1.27 (0.93, 1.74)	0.135	1.14 (0.84, 1.55)	0.412
rs7202877	*BCAR1*	16	75247245	T	0.88	0.85 (0.59, 1.23)	0.393	0.94 (0.61, 1.45)	0.794	0.93 (0.61, 1.42)	0.738	0.69 (0.43, 1.11)	0.129
rs1359790	*SPRY2*	13	80717156	G	0.77	0.88 (0.66, 1.18)	0.397	0.90 (0.64, 1.26)	0.522	0.83 (0.59, 1.17)	0.284	0.99 (0.70, 1.40)	0.946
rs12242953	*VPS26A*	10	70865342	G	0.93	1.25 (0.74, 2.11)	0.402	1.25 (0.69, 2.28)	0.461	1.18 (0.63, 2.20)	0.613	1.44 (0.76, 2.72)	0.259
rs2075423	*PROX1*	1	214154719	G	0.62	0.89 (0.69, 1.17)	0.411	0.85 (0.62, 1.18)	0.332	0.88 (0.65, 1.21)	0.432	0.99 (0.72, 1.36)	0.945
rs6878122	*ZBED3*	5	76427311	G	0.28	1.12 (0.85, 1.48)	0.416	1.31 (0.94, 1.82)	0.115	1.18 (0.84, 1.65)	0.342	0.97 (0.70, 1.35)	0.868

rs1111875	*HHEX/IDE*	10	94462882	C	0.64	0.90 (0.68, 1.17)	0.425	0.87 (0.63, 1.19)	0.383	0.96 (0.70, 1.32)	0.816	0.88 (0.64, 1.21)	0.435
rs1552224	*ARAP1 (CENTD2)*	11	72433098	A	0.86	1.18 (0.79, 1.76)	0.425	1.47 (0.93, 2.32)	0.095	1.12 (0.69, 1.80)	0.643	0.93 (0.57, 1.53)	0.781
rs10758593	*GLIS3*	9	4292083	A	0.46	0.90 (0.70, 1.17)	0.433	1.04 (0.77, 1.40)	0.793	0.95 (0.71, 1.28)	0.740	0.79 (0.58, 1.08)	0.138
rs11651052	*HNF1B (TCF2)*	17	36102381	A	0.43	1.10 (0.86, 1.42)	0.449	1.15 (0.86, 1.55)	0.343	1.02 (0.75, 1.39)	0.887	1.20 (0.88, 1.63)	0.257
rs11717195	*ADCY5*	3	123082398	T	0.84	1.15 (0.80, 1.65)	0.461	1.36 (0.90, 2.06)	0.141	1.11 (0.71, 1.73)	0.644	0.95 (0.61, 1.50)	0.842
rs2796441	*TLE1*	9	84308948	G	0.64	1.10 (0.85, 1.44)	0.461	1.35 (0.99, 1.84)	0.054	0.92 (0.66, 1.28)	0.629	1.02 (0.73, 1.41)	0.926
rs5215	*KCNJ11*	11	17408630	C	0.38	0.91 (0.70, 1.18)	0.462	1.06 (0.78, 1.44)	0.710	0.84 (0.62, 1.15)	0.275	0.81 (0.59, 1.11)	0.190
rs7756992	*CDKAL1*	6	20679709	G	0.32	0.91 (0.70, 1.19)	0.478	0.92 (0.67, 1.25)	0.582	0.84 (0.61, 1.16)	0.301	0.96 (0.70, 1.32)	0.807
rs7955901	*TSPAN8/LGR5*	12	71433293	C	0.43	0.91 (0.70, 1.18)	0.488	0.88 (0.64, 1.21)	0.435	0.93 (0.68, 1.27)	0.648	0.90 (0.65, 1.24)	0.502
rs1496653	*UBE2E2*	3	23454790	A	0.86	0.88 (0.62, 1.26)	0.493	1.10 (0.73, 1.65)	0.641	0.86 (0.55, 1.33)	0.489	0.76 (0.48, 1.19)	0.225
rs2943640	*IRS1*	2	227093585	C	0.65	0.91 (0.70, 1.19)	0.510	0.88 (0.63, 1.23)	0.453	0.90 (0.65, 1.24)	0.508	0.96 (0.70, 1.33)	0.813
rs2854275	*HLA*	6	32628428	C	0.92	0.86 (0.54, 1.36)	0.513	0.91 (0.52, 1.60)	0.744	0.83 (0.47, 1.46)	0.519	0.80 (0.45, 1.42)	0.446
rs17867832	*GCC1*	7	126996837	T	0.91	0.87 (0.56, 1.35)	0.523	0.88 (0.52, 1.49)	0.644	0.73 (0.42, 1.27)	0.271	1.12 (0.64, 1.95)	0.686
rs17168486	*DGKB*	7	14898282	T	0.18	1.11 (0.80, 1.52)	0.533	1.14 (0.78, 1.66)	0.488	1.19 (0.81, 1.74)	0.379	1.10 (0.76, 1.59)	0.629
rs7845219	*TP53INP1*	8	95937502	T	0.54	0.92 (0.72, 1.19)	0.537	0.98 (0.72, 1.33)	0.891	0.92 (0.67, 1.25)	0.587	0.84 (0.62, 1.13)	0.243
rs7177055	*HMG20A*	15	77832762	A	0.70	1.06 (0.80, 1.41)	0.672	1.07 (0.77, 1.50)	0.679	0.91 (0.64, 1.29)	0.610	1.19 (0.87, 1.64)	0.280

rs12970134	*MC4R*	18	57884750	A	0.25	1.06 (0.79, 1.42)	0.701	0.96 (0.68, 1.35)	0.810	1.26 (0.87, 1.82)	0.221	0.99 (0.70, 1.40)	0.963
rs9273363	*HLA*	6	32626272	A	0.21	0.94 (0.68, 1.30)	0.705	0.85 (0.57, 1.27)	0.432	1.06 (0.72, 1.57)	0.773	0.86 (0.59, 1.27)	0.453
rs13389219	*GRB14*	2	165528876	C	0.62	1.05 (0.81, 1.36)	0.715	1.02 (0.74, 1.40)	0.897	1.12 (0.82, 1.54)	0.472	0.98 (0.72, 1.34)	0.909
rs849135	*JAZF1*	7	28196413	G	0.54	1.04 (0.81, 1.34)	0.735	1.14 (0.85, 1.53)	0.379	1.05 (0.78, 1.42)	0.750	1.02 (0.75, 1.37)	0.920
rs3802177	*SLC30A8*	8	118185025	G	0.73	1.05 (0.79, 1.40)	0.737	1.29 (0.93, 1.80)	0.129	1.00 (0.69, 1.44)	0.980	1.06 (0.74, 1.50)	0.756
rs4458523	*WFS1*	4	6289986	G	0.63	0.96 (0.73, 1.25)	0.746	1.07 (0.78, 1.48)	0.668	0.88 (0.63, 1.24)	0.470	0.87 (0.63, 1.20)	0.388
rs10830963	*MTNR1B*	11	92708710	G	0.33	1.03 (0.79, 1.34)	0.825	1.07 (0.77, 1.49)	0.666	1.00 (0.73, 1.36)	0.986	1.17 (0.85, 1.62)	0.333
rs17301514	*ST64GAL1*	3	186613409	A	0.16	0.96 (0.69, 1.34)	0.827	0.99 (0.67, 1.45)	0.957	0.92 (0.61, 1.38)	0.680	0.96 (0.65, 1.41)	0.821
rs10811661	*CDKN2A/B*	9	22134094	T	0.87	0.96 (0.65, 1.41)	0.831	0.93 (0.60, 1.44)	0.752	0.77 (0.49, 1.22)	0.265	1.22 (0.77, 1.92)	0.395
rs9936385	*FTO*	16	53819169	C	0.46	1.02 (0.79, 1.31)	0.892	0.93 (0.69, 1.25)	0.624	1.09 (0.82, 1.45)	0.544	1.09 (0.81, 1.47)	0.555
rs4812829	*FTITM2/R3 HDML/HN F4A*	20	42989267	A	0.20	1.02 (0.74, 1.40)	0.905	0.91 (0.63, 1.32)	0.630	1.06 (0.72, 1.56)	0.767	1.09 (0.74, 1.60)	0.665
rs12427353	*HNF1A (TCF1)*	12	121426901	G	0.84	0.98 (0.70, 1.39)	0.920	1.10 (0.73, 1.67)	0.641	0.96 (0.63, 1.44)	0.834	0.88 (0.58, 1.35)	0.556
rs2447090	*SRR*	17	2298974	A	0.65	1.01 (0.77, 1.32)	0.949	1.00 (0.73, 1.39)	0.976	1.03 (0.74, 1.42)	0.875	1.06 (0.77, 1.47)	0.718
rs10401969	*CILP2*	19	19407718	C	0.07	0.99 (0.61, 1.61)	0.958	1.19 (0.66, 2.15)	0.567	0.89 (0.49, 1.61)	0.696	0.96 (0.55, 1.68)	0.888
rs12571751	*ZMIZ1*	10	80942631	A	0.53	0.99 (0.77, 1.29)	0.962	1.21 (0.89, 1.66)	0.226	0.94 (0.69, 1.27)	0.674	0.94 (0.69, 1.28)	0.701
rs1801282	*PPARG*	3	12393125	C	0.85	1.01 (0.71, 1.43)	0.971	1.02 (0.68, 1.54)	0.917	0.97 (0.63, 1.48)	0.879	0.96 (0.64, 1.45)	0.844

Association of SNPs with MOD cluster as case group, p-values, OR and 95% CI were calculated using logistic regression adjusted for sex and age. Chr – chromosome, BP – base pair, RAF – risk allele frequency. Number of individuals in the analyses IROD2 vs. all clusters: N=837, IROD2 vs. IROD1: N=357, IROD2 vs. SIDD: N=355, IROD2 vs. MARD: N=415. *significant after adjustment for the multiple testing using Bonferroni correction (< 0.05).

2.2 Supplemental Table 3: Clinical characteristics and prevalence of macro- and microvascular complications in the entire DOLCE cohort.

Phenotype	Entire cohort
N, (men, %)	2140 (35%)
Age at visit, years	59.4 (11.3)
Age at onset of diabetes, years	52.5 (11.9)
Diabetes duration, years	6.9 (7.3)
HbA1c, %	8.5 (2.1)
HbA1c, mmol/mol	69.6 (22.9)
BMI, kg/m2	30.8 (5.4)
Waist, cm	98.4 (13.4)
HOMA2-B	71 (46.2)
HOMA2-IR	2.3 (1.3)
C-peptide, nmol/l	0.9 (0.5)
Without treatment, %	26.5%
Tablets, %	47.9%
Insulin, %	19.9%
Tablets and insulin, %	5.7%
Retinopathy, %	3.4%
CKD, %	20.8%

Neuropathy, %	64.3%
CVD, %	12.3%

Data are mean ± (SD). PDR – proliferative diabetic retinopathy. CKD – chronic kidney disease. CVD - cardiovascular disease.

2.3 Supplemental Table 4. Risk of macro- and microvascular complications in new-onset adult diabetes group in different clusters relative to MARD cluster (n=887).

Complications	SAID		SIDD		IROD1		IROD2	
	OR (95% CI)	p-value	OR (95% CI)	p-value	OR (95% CI)	p-value	OR (95% CI)	p-value
PDR**	-	-	-	-	-	-	-	-
CKD	5.78 (2.15, 15.54)	5.1×10^{-4}*	2.87 (1.63, 5.06)	6.7×10^{-4}*	1.01 (0.58, 1.75)	0.98	-	-
Neuropathy	6.90 (3.08, 15.49)	2.8×10^{-6}*	3.23 (1.98, 5.27)	2.7×10^{-6}*	1.14 (0.76, 1.7)	0.53	2.22 (1.07, 4.58)	0.03
CVD	-	-	0.55 (0.2, 1.54)	0.26	1.12 (0.62, 2.04)	0.71	0.59 (0.21, 1.64)	0.32

PDR – proliferative diabetic retinopathy. CKD – chronic kidney disease. CVD - cardiovascular disease. Odds Ratios (OR), 95% confidence intervals (95% CI) and p-values were calculated using logistic regression adjusted for sex, age, and diabetes duration. There were n=3 cases with CKD in MOD, n=3 with PDR in MARD cluster, and n=1 case of CVD in SAID cluster and therefore these calculations of OR were not performed.

*Significant after adjustment for the multiple testing using FDR (< 0.05).

**Data for PDR in individuals from SAID cluster was available for 92 individuals from the group with long-term and only for 46 individuals in the group with new-onset adult diabetes.
